# Supplementary material for: Regionalized Protein Localization Domains in the Zebrafish Hair Cell Kinocilium
Source: J Dev Biol. 2023 Jun 16;11(2):28. doi: 10.3390/jdb11020028 (PMC10299642; doi:10.3390/jdb11020028)
Supplement: Supplementary file 1 [file jdb-11-00028-s001.zip › jdb-2216713-Figures_S1-8.pdf]

Journal of
Developmental Biology

MDPI

Erickson et al. Figures S1-8

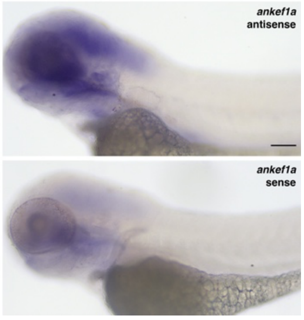

Figure S1. Wholemount mRNA in situ hybridization (ISH) for *ankef1a* on 3 day post-fertilization zebrafish larvae using antisense (top) and sense (bottom) DIG-labeled probes targeted to nucleotides 1912-2687 of ENSDART00000153006.2. Primers used to generate the *ankef1a* ISH probe templates are detailed in Table S1. Scale bar = 100 µm.

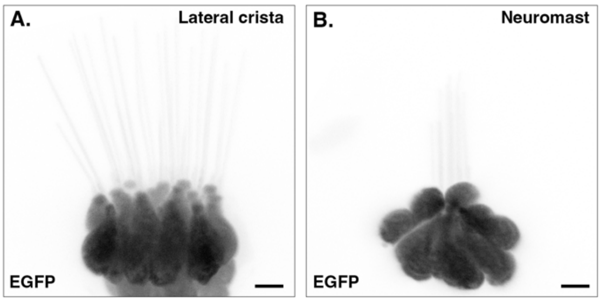

Figure S2. EGFP localization in hair cells of a lateral crista (**A**) and lateral line neuromast (**B**) in a *Tg(myo6b:EGFP)vo68* transgenic zebrafish at 5 days post-fertilization. Scale bars = 5 µm.

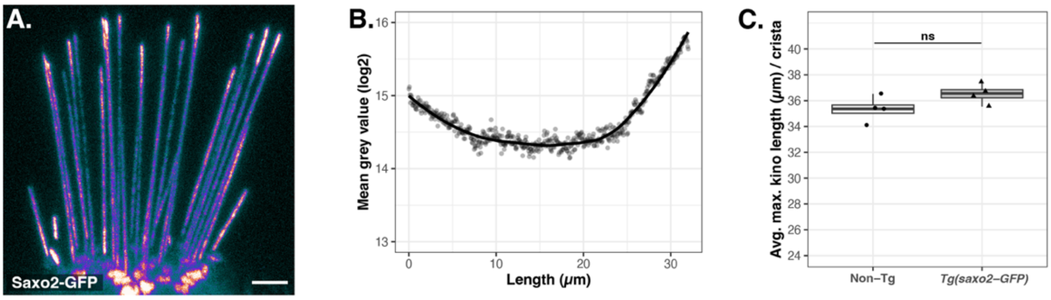

Figure S3. Saxo2-GFP kinocilial distribution and effect on kinocilia height. **A.** Pixel intensity-encoded image of Saxo2-GFP in the kinocilia of lateral crista hair cells at 5 days post-fertilization (dpf). Brighter colours indicate higher fluorescence intensity. **B.** Scatter plot of averaged mean grey values (log2) for fluorescence intensity from kinocilia in the lateral cristae of Saxo2-GFP transgenics (n = 16 kinocilia from 4 individuals at 5 dpf). Position 0 µm is from the proximal region near the base of kinocilia and position 32 µm is the distal-most tip. **C.** Boxplots of maximum kinocilia length in the lateral crista of *saxo2-GFP* transgenics and their non-transgenic siblings at 5 dpf. Data points are the average length of the five tallest kinocilia from an individual larva. Two-tailed Welch's t-test statistics: non-Tg, n = 4 larvae, mean = 35.3 µm; *Tg(saxo2-GFP)*, n = 4, mean = 36.5 µm; t = -1.8712, df = 5.5674, p = 0.1143. Scale bar is 5 µm in A.

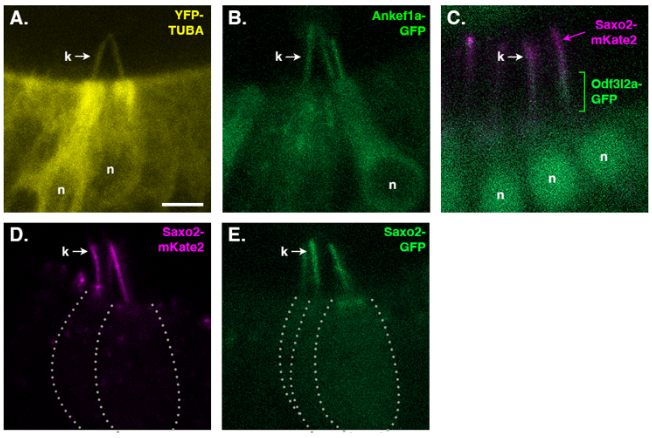

Figure S4. Transgenic protein localization in hair cells of the anterior macula: **A.** YFP-TUBA; **B.** Ankef1a-GFP; **C.** Odf3l2a-GFP with Saxo2-mKate2; **D.** Saxo2-mKate2; **E.** Saxo2-GFP. All images are from 1-day post-fertilization (dpf) larvae, except panel C which is at 5 dpf due to low fluorescence intensity for Odf3l2a-GFP at 1 dpf. Approximate cell outlines are indicated by dashed lines in panels D and E. k = kinocilium, n = nucleus. Scale bar = 5 µm, applies to all images.

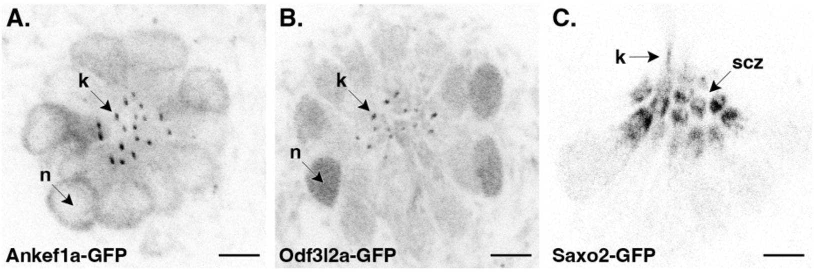

Figure S5. Ankef1a-GFP (**A**), Odf3l2a-GFP (**B**), and Saxo2-GFP (**C**) localization patterns in the soma of neuromast hair cells (top-down views). k = kinocilium, n = nucleus, scz = subcuticular zone. Scale bars = 5 µm.

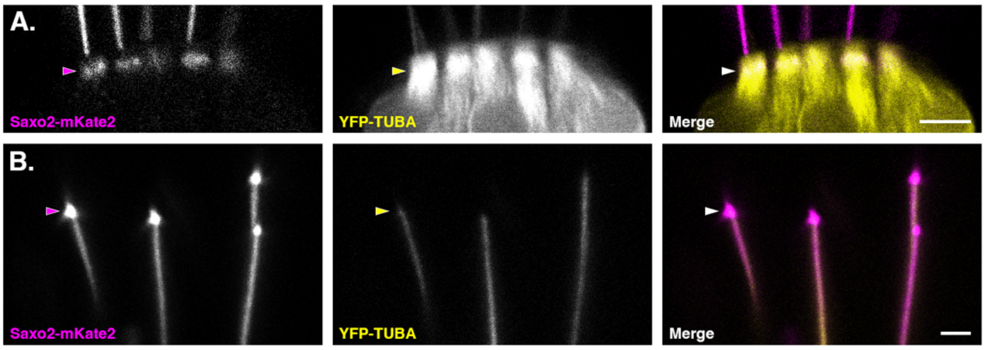

Figure S6. Details of Saxo2-mKate2 and YFP-TUBA co-localization in the subcuticular zone (**A**) and at the distal tip of kinocilia (**B**) in the lateral crista at 6 days post fertilization. Scale bar is 5 µm in A and 2 µm in B.

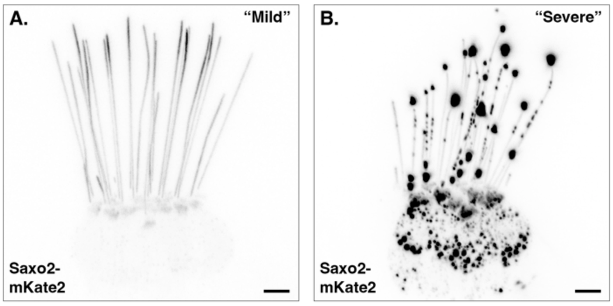

Figure S7. Comparison of fluorescent protein intensity levels in Saxo2-mKate2 "mild" (**A**) and "severe" (**B**) transgenic lines at 6 days post-fertilization. The image in panel B was captured at 88% of the gain of that in panel A, thus slightly underrepresenting the true intensity difference between the two lines. Scale bars = 5 µm.

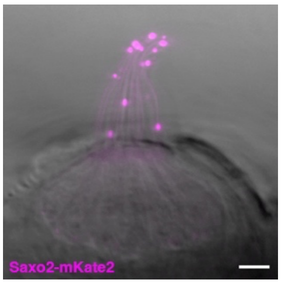

Figure S8. Example of the "severe" phenotype in a neuromast of a *saxo2-mKate2* transgenic at 5 days post-fertilization. Scale bar = 5 µm.

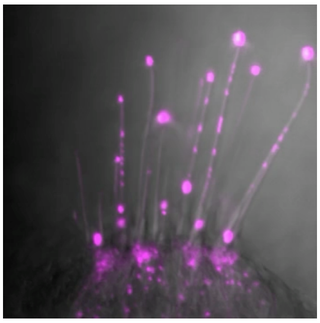

Video S1. Movement of Saxo2-mKate2 positive punctae in the kinocilia of lateral crista hair cells in a 5 dpf zebrafish larva. Note the bi-directional motion of the puncta on the left. Video info: 20 frames over 60 sec, played back at 7 fps, depth of 4.7 µm, frame dimension 40x40 µm.
